# Supplementary material for: A New Surgical Site Infection Risk Score: Infection Risk Index in Cardiac Surgery
Source: J Clin Med. 2019 Apr 9;8(4):480. doi: 10.3390/jcm8040480 (PMC6517895; doi:10.3390/jcm8040480)
Supplement: Supplementary file 1 [file jcm-08-00480-s001.pdf]

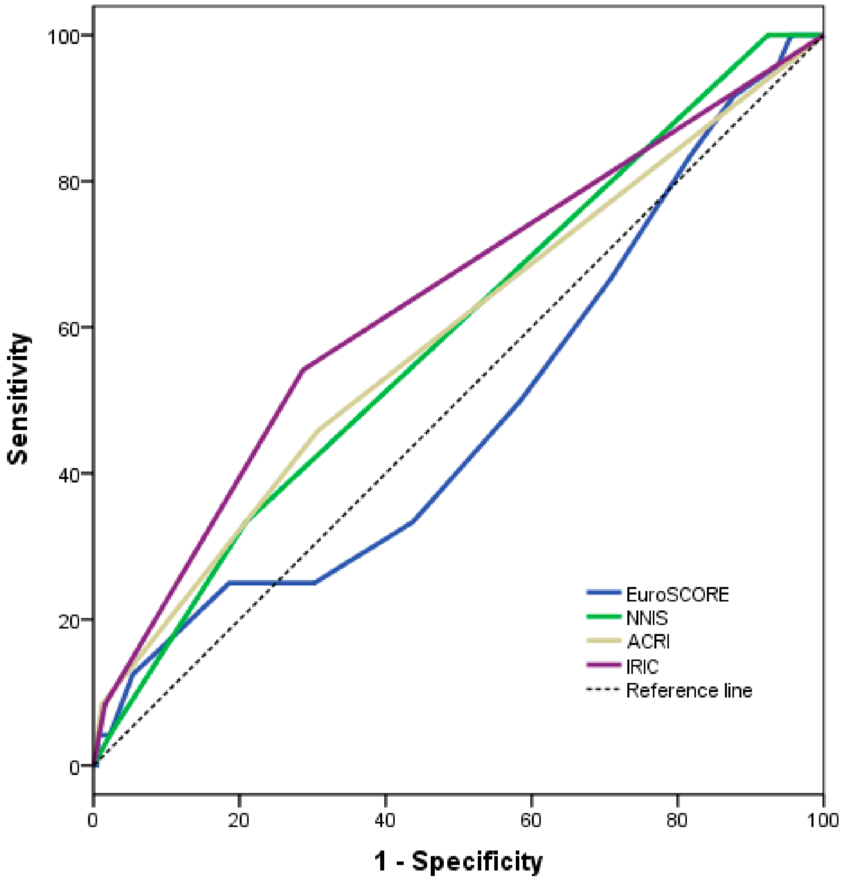

	aROC	Standard Error	p	95%CI
**EuroSCORE**	0.483	0.062	0.778	0.361–0.605
**ACRI**	0.609	0.057	0.047	0.498–0.720
**NNIS**	0.605	0.052	0.054	0.504–0.707
**IRIC**	0.657	0.054	0.004	0.550–0.764

Supplementary Figure S1. Comparisons of aROC corresponding to IRIC, NISS, EuroSCORE and ACRI to evaluate differences between these scoring systems (test sample). ACRI, Australian Clinical Risk Index; aROC, area under the Receiver-Operating Characteristic curve; CI, confidence interval; IRIC, Infection Risk Index in Cardiac surgery; NNIS, National Nosocomial Infections Surveillance.

Supplementary Table S1. Patients in the training and the test sample.

	**Training Sample 2010–2014**	**Test Sample 2015–2017**	***p***
	n = 1299	*n* = 722	
Age in Years (mean)	70.52	71.13	0.170
Males (%)	56.7	59.1	0.281
Diabetes (%)	26.6	28.5	0.376
Obesity or >30kg/m2 (%)	5.1	7.9	0.015
Peripheral Artery Disease (%)	7.2	9.2	0.190
EuroSCORE (mean)	5.86	6.00	0.379
ASA score ≥III (%)	88.1	87.1	0.391
Clean surgery (%)	91.3	93.1	0.111
Duration of surgery in min (mean)	282.19	239.99	0.000
Aortic clamping time in min (mean)	67.61	74.63	0.000
Total CPB in min (mean)	95.96	103.59	0.004
Reoperation for bleeding (%)	4.5	7.3	0.011
Surgical Site Infection (%)	4.6	4.0	0.573

ASA, American Society of Anesthesiologists; CABG, coronary artery bypass graft; CARD, cardiac valve surgery; CPB, cardiopulmonary bypass.
